# Barriers and facilitators for guideline adherence in diagnostic imaging: an explorative study of GPs’ and radiologists’ perspectives

**DOI:** 10.1186/s12913-018-3372-7

**Published:** 2018-07-16

**Authors:** Ann Mari Gransjøen, Siri Wiig, Kristin Bakke Lysdahl, Bjørn Morten Hofmann

**Affiliations:** 1 Department of Health sciences in Gjøvik, Norwegian University of Science and Technology in Gjøvik (NTNU), Teknologiveien 22, 2815 Gjøvik, Norway; 2 Faculty of Health Studies, University of Stavanger, Kjell Arholmsgate 41, 4036 Stavanger, Norway; 3 Faculty of Health Sciences, OsloMet – Oslo Metropolitan University, Pilestredet 46, 0167 Oslo, Norway; 4 Department of Optometry, Radiography and Lighting Design, Faculty of Health Sciences, University of South-Eastern Norway, PO Box 235, 3603 Kongsberg, Norway; 5 Center for medical ethics, University of Oslo, PO Box 1130 Blindern, 0318 Oslo, Norway

**Keywords:** Diagnostic imaging, Barriers, Facilitators, Guideline adherence, Unwarranted imaging

## Abstract

**Background:**

Diagnostic imaging has been a part of medicine for the last century. It has been difficult to implement guidelines in this field, and unwarranted imaging has been a frequent problem. Some work has been done to explain these phenomena separately. Identifying the barriers to and facilitators of guideline use has been one strategy. The aim of this study is to offer a more comprehensive explanation of deviations from the guideline by studying the two phenomena together.

**Methods:**

Eight general practitioners and 10 radiologists from two counties in Norway agreed to semi-structured interviews. Topics covered in the interviews were knowledge of the guideline, barriers to and facilitators of guideline use, implementation of guidelines and factors that influence unwarranted imaging.

**Results:**

Several barriers to and facilitators of guideline use were identified. Among these are lack of time, pressure from patients, and guidelines being too long, rigid or unclear. Facilitators of guideline use were easy accessibility and having the guidelines adapted to the target group. Some of the factors that influence unwarranted imaging are lack of time, pressure from patients and availability of imaging services.

**Conclusion:**

There are similarities between the perceived barriers for guideline adherence and the perceived factors that influence unwarranted imaging. There may be a few reasons that explains the deviation from guidelines, and the amount of unwarranted imaging.

**Electronic supplementary material:**

The online version of this article (10.1186/s12913-018-3372-7) contains supplementary material, which is available to authorized users.

## Background

Diagnostic imaging has been a part of medicine since the late nineteenth century, and unwarranted imaging has increased extensively over the last decades [1]. The proliferation of imaging facilities has lead to an increased awareness of unnecessary imaging. The inappropriate use of imaging is estimated to be from 10% up to 50%. [2–7]. Aspects contributing to the number of unwarranted images are, among others, defensive medicine, duplicate examinations, doing examinations too early and doing the wrong examinations, i.e. aspects relating to organization of health services and health personnel [8].

There are also many aspects influencing the decision to refer a patient to diagnostic imaging, and include patients’ expectations, the accessibility of diagnostic imaging, the patients’ symptoms and the number of visits the patient has made with the General Practitioner, i.e. aspects relating to the patient, and accessibility of diagnostic imaging services [9–12]. Together with aspects relating to technology (such as lack of equipment) [13], and geography and location (geographical variations) [14], this provides a comprehensive explanation of unwarranted imaging on the individual, organizational and national levels. One possible way to reduce unnecessary imaging is to implement and enforce guidelines that ensure best practices and equal treatment of all patients [15]. Guidelines have been reported to decrease needless imaging [16–18] and the amount of diagnostic imaging overall [18–21], although this effect is not always apparent. However, enforcing guidelines can be difficult, and adherence to guidelines is an identified problem in health care [22] and especially in radiology [23].

Some of the barriers to guideline use are the target groups’ lack of awareness of, and familiarity with the guideline, time constraints, a lack of motivation with the user, patient expectations, disagreement with the guideline, and organizational constraints [9, 13, 24, 25]. Conversely, facilitators for the use of guidelines include support from leaders, senior staff accepting and using the guideline, and having enough time to follow the recommendations given in the guideline [24]. Radiologists have reported the same problems with guideline implementation that GPs do such as the aversion to cookbook medicine, resistance to guidelines, and lack of education on the guidelines [26].

There are guidelines for medical imaging for many fields and in many countries. Although the guidelines are related, each guideline is attuned to its local context. Context is also important for adherence to guidelines. Accordingly, the objective of this study is to identify barriers and facilitators to guideline implementation and adherence in Norway, as well as investigating factors contributing to unwarranted imaging.

In order to be context specific, this study relates to a guideline published by the Norwegian Directorate of Health in 2014. This guideline is aimed at GPs, and contains recommendations regarding when to use the four most common radiological modalities (conventional radiography, computed tomography, magnetic resonance imaging and ultrasound) for diagnosis of disease in the extremeties, spine, hip and pelvis. [27]. One of the explicit goals of this guideline is to make practice more coherent, ensuring equal treatment for all patients [28].

This study is an exploratory study facilitators of and barriers to guideline implementation and adherence in Norway. It also investigates the factors that contribute to unwarranted imaging.

GPs are included in this study because they are the primary target group for the guideline.. Radiologists are also included as they are obliged by law to assess the justification of a referred procedure, and therefore need to have knowledge on the latest evidence regarding optimal examinations. In this context the decision of performing a radiological procedure can be seen as a collaboration between the referrer and the assessor/executer of the procedure.

The research questions in this study are as follows: *What are the perceived facilitators of and barriers to adherence to Norway’s national guidelines for diagnostic imaging in non-traumatic musculoskeletal disorders (MSDs) by GPs and radiologists? Which factors do Norway’s GPs and radiologists perceive as contributing to the requisition and execution of unwarranted diagnostic imaging?*

There is a want of qualitative studies regarding these subjects including both GPs and radiologists, both of which need to have knowledge of guidelines regarding diagnostic imaging. By including both groups similarities and differences in perceived barriers and facilitators for guideline use can be identified, helping the development and implementation of shared and optimized guidelines for these groups. Posing these questions both to GP’s and to radiologists is unique to this study, and lessons learned from the Norwegian setting with the Norwegian Musculoskeletal Guideline (NMSG) may be highly relevant in other contexts.

The two research questions of the study are closely connected. Several factors contributing to unwarranted imaging are linked to the barriers to guideline adherence. Therefore, we hope to give a more comprehensive explanation of why guidelines are not followed and why unwarranted imaging occur by investigating both phenomena.

## Methods

### Design

This study is the first, exploratory, phase of a project that aims to improve the health service by effective implementation of the NMSG. This particular guideline is of interest for this project since it is relatively new, and not actively implemented.

To explore the factors contributing to both the appropriate and inappropriate use of diagnostic imaging and to the barriers to and facilitators of guideline adherence, qualitative interviews was conducted with eight GPs, eight board-certified radiologists, and two registered trainees in radiology (registrars). in two Norwegian couties from October 2016 to March 2017. AMG conducted all interviews. As a trained radiographer, she has first hand knowledge of the field, and the Norwegian context.

### Participants and data collection.

Eight GPs and 10 radiologists agreed to participate in this study. 6 of the 8 GPs were female, and 5 of the 8 worked in county A.8 of the 10 radiologists were male, and about 8 of the 10 worked in county B. The recruitment of participants were initiated through telephonic contact, or by e-mail to their place of work. The inclusion criteria for the GPs was that they were in clinical practice in a group setting, for radiologists that they were practicing at either hospitals or private institutions. Secondly there were held 8 short informational meetings where the participants received further information regarding the study, and could decide whether they wished to participate.

Data were collected through qualitative, semi-structured individual interviews. This was to prevent leading questions from being asked, and still ensure that the topics of interest were covered. The interviews were conducted in two counties that were similar in their demographics and in the amount of diagnostic imaging performed, but different enough to ensure variation in the selection. General practitioners and radiologists were selected because the former are responsible for referring patients with musculoskeletal complaints to diagnostic imaging, and the latter are responsible for determining if the referral is justified.

All interviews were conducted at the participant’s workplace. Most of the interviews lasted approximately 30 min; a few were 20 or 40 min long. An interview guide where the themes were inspired by Human Factors Theory [29] and the Consolidated Framework for Implementation Research (CFIR) [30] was developed. This covered the participants’ knowledge of theNMSG; and in general what constituted a guideline they would want to use, what made it less likely that they wanted to use a guideline, implementation of guidelines, as well as which factors they saw as contributing to patients being referred to diagnostic imaging. The Interview guide explains this in more detail (see Additional file 1).

### Data analysis

The data material consists of 18 individual semi-structured interviews. All interviews were tape-recorded and transcribed verbatim. The data was analyzed using systematic text condensation consisting of the following steps: (1) establishing an overview of the data material and identifying preliminary themes; (2) identifying and sorting units of meaning into code groups; (3) condensing the contents of each of the coded groups into subgroups; and (4) summarizing the contents of each code group into categories, in order to generalize descriptions and concepts [31].

All authors contributed in reading the interviews and identifying the preliminary themes in step 1, then AMG conducted steps 2–4 of the analysis in close collaboration with all authors to avoid bias. Two themes relevant to the research questions are reported in this article: the barriers to and facilitators of guideline adherence, and the contributing factors to unwarranted imaging.

### Ethical approval

The study has ethical approval from the Norwegian Social Science Data Services (NSD) (Ref. 48,267, 06 May 2016). Changes were made to the interview guide after a pilot study, also approved by NSD. Participation was voluntary and written informed consent was obtained from all participants. Data were anonymized and securely stored.

## Results

In total 8 radiologists and 2 radiological fellows working in two different public hospitals, and 8 GPs working in 7 different GP offices participated in the study. For an overview of participant characteristics, see Table 1.

**Table 1:** Overview of participants, the gender distribution and characteristics of their workplace

	Profession	Gender distribution	Sites visited	Total number of participants
County A	5 GPs, 2 radiologists	5 females, 2 males	1 hospital, 5 GP offices, all group settings	7
County B	3 GPs, 6 radiologists, 2 radiological fellows	3 females, 8 males	1 hospital, 2, GP offices, all group settings	11

The analysis generated eight categories in total. Four of these categories are related to the barriers to and facilitators of guideline adherence and the other four pertained to factors influencing the overuse of diagnostic imaging. Of the four categories relating to barriers and facilitators of guideline adherence two consider the practicality and usability of the guidelines, and the last two consider the usefulness of guidelines and how the knowledge basis of the guideline influences adherence. Of the four categories relating to factors influencing unwarranted imaging, three address the pressures and incentives from outside the medical centers and departments, and the last category addresses the usefulness of diagnostic imaging. All eight categories incorporate viewpoints from both GPs and radiologists.

In general the NMSG were somewhat known (5/8 GPs and 4/10 radiologists reported having knowledge of the guideline), however it was not widely used (2/8 GPs and none of the radiologists reported trying to have used the guideline). There are several guidelines that were mentioned by the participants in addition to the national guideline on diagnostic imaging in non-traumatic musculoskeletal diseases. All of these guidelines were developed by the Norwegian Directory of Health in cooperation with specialists in the relevant fields. They are all freely accessible online; however, they main forms of implementation used are passive methods, such as publishing and postal dissemination.

### Guidelines need to be practical and adapted to the target group

All GPs and radiologists reported easy access to the guideline as a facilitator of guideline adherence, and 5 GPs and 7 radiologists perceived adaption to the target group as a facilitator.. There was no consensus on the form that access should take; where the divide was between having guidelines as booklets (2 GPs and 2 radiologists preferred booklets) or online (majority of both GPs and radiologists preferred to find them online). One GP expressed the benefit of having booklets: “*Yeah, you will be unsure sometimes anyway, and it’s easy to look it up in a booklet.*” Easy access was an important facilitator, since there usually was little time to look up guidelines as soon they were needed. Only one GP noted that Computerized Physician Order Entry (CPOE) could be a facilitator for guideline use.

At the same time, guidelines being too difficult to find or too long to read were seen as barriers to guideline use, this was reported by the majority of both GPs and radiologists. Barriers that were related to the guidelines adaption toward the target group was that guidelines could be too rigid, as reported by a radiologist: “*Yeah, that they are too rigid, and do not show much discretion when considering age for example*.” Radiologists were most likely to complain that the guidelines failed to consider natural physiological and anatomical changes that occur with age, or the differences between the sexes (2 radiologists reported this, vs 1 GP).

### Experienced usefulness of the guidelines

Something increasing the perceived usefulness of guidelines reported by the GPs alone, was the fact that everyone relied on the same guidelines, increasing the confidence of not making decisions leading to unwarranted examinations or treatment. The radiologists complained of the slow publication of the guidelines, which were obsolete by the time they were received by the radiological departments. This made the guidelines seem unnecessary, and less likely to be used. Lastly, radiologists disliked what they perceived to be a formality of guidelines; radiologists preferred more local protocols and informal knowledge exchange to formal, national guidelines. Local protocols and informal knowledge sharing were perceived as more up to date and therefore better suited to radiological departments.

### Knowledge basis must be clear and accepted by the target group

Radiologists and GPs all commented that the knowledge basis must be clear in a guideline. This clarity encouraged the use of guidelines, making them more likely to be used when a GP was unsure of what kind of exam or treatment was needed. Conversely, one GP reported disagreement with a guideline as a significant barrier for use:

There was for example a new guideline regarding maternity care, which I have not used because I disagreed with it. It said that there was no professional justification for performing a gynecological examination, and you could say that well, how often is it justified to perform a gynecological examination at all without any special symptoms?

In this example the GP draws on experience from another guideline (the national guideline for maternity care for healthy women and newborns carried to term) which underscores they were reflecting in guidelines in general.

Two radiologists similarly complained that people who were not familiar with the clinical environment had developed the guidelines. This can again lead to disagreement with the guideline, or the feeling that it does not apply to the setting where it is intended to be used.

### Too many guidelines diminish use

Two related factors reported by both groups shows why too many guidelines are a deterrent to their use, reported by 4 GPs and 3 radiologists. The first factor is that there are too many guidelines and not enough time to keep up with them. According to one GP: “*There are so many papers and guidelines you have to relate to, you know?*” The other factor is that some guidelines are not high priority because there are so many guidelines in total. At the same time the guidelines are still known, and the users will try to bring them up and talk about them during staff meetings, as reported by this GP: “*We read them, and try to talk about them in meetings, but many of them are probably not used*.” The guidelines that tend to not be prioritized are those that are either unnecessary or so obvious that they do not need to be written.

### Higher demand to exclude disease

Some factors influencing unwarranted imaging were found in both groups. One of those factors was increased patient demand, especially regardingreferrals and the pressure the patient puts on the GP, reported by the majority of both GPs and radiologists. A second factor was the need for the GP to exclude any uncertainty of a diagnosis, as reported by this GP:

When someone comes in with a long-lasting cough so you auscultate and think it may be a pneumonia, before you would think it probably is a pneumonia. But now, for safety’s sake, we take an x-ray to make sure the diagnosis is correct*.*

This was addressed in relation to performing the examination and the fear of misdiagnosing a serious illness like cancer. Another factor in relation to the need for excluding any uncertainty of a diagnosis is a greater need for documentation as experienced by the majority of GPs. This is insistence on documentation was found in from patients, as well as from other medical professions and even non-medical agencies, where the Norwegian Labour and Welfare services (NAV) was especially mentioned. Lastly, three radiologists described a change in the way referrals were being written. According to the radiologists, there is more interest in ruling out disease than in confirming a diagnosis than before. Referrals were also less precisely formulated than before, and therefore harder to justify.

### Diagnostic imaging has high utility

Both GPs and radiologists noted the high utility of diagnostic imaging, even though from their respective perspectives as GPs and radiologists. For GPs this meant a shift in modality: “*Where you could only use plain radiography before, you use MRI because you have gotten used to it.*” This shows that GPs have grown accustomed to referring to modalities heavier on both information and use of resources, and that they are viewed as highly useful. One GPs also addressed a lack of options for examinations or treatment that could be done for these patients (for example long queues for physiotherapy, and MRI being the most informative imaging modality). A majority of both GPs and radiologists also stated that diagnostic imaging could increase patient safety, especially in relation to surgery where a surgeon could get a detailed overview of a patient’s condition before beginning the procedure.

### Political and economic gain

According to one radiologist “*The hospital will be paid more for performing outpatient examinations, the patient is more pleased because we do a lot of tests and the hospital’s statistics will look good, both economically and purely numerically*.” This comment was seen in relation to the demand of production in the radiology department and the hospital wanted to increase the number of outpatients for financial reasons. Another factor related to the economic benefit of diagnostic imaging is the privatization of the health care services, as expressed by this radiologist:

So it’s many institutions who want customers right, I think that’s an essential point here, the privatization of health care and running a health business rather than healthcare. That’s scary, because when profit decides what you do, you will try to recruit people to examine.

The majority of both groups claimed that private institutions have a higher demand for profits, making a higher demand for production, which again influences unwarranted imaging. Four radiologists also viewed the organizational structure as leading to excessive imaging, moving patients through the system so as not to overcrowd the emergency rooms and wards.

When it comes to political gain, a minority of both groups reported unwarranted imaging to be closely related to election campaigns. The experience was that politicians made promises regarding the availability of services that the health care workers were left to implement them. These promises could, for example, be shorter waiting time to get an appointment with a GP, or to get an appointment for diagnostic imaging.

### Uncertainty and time pressure

Two factors regarding uncertainty and time pressure were reported; one from the radiologists alone, and the other from both the radiologists and the GPs. Two radiologists reported that the GPs needed to be more up to date on the indications for the different types of diagnostic imaging. These indications were not always used correctly, leading to even more possibly unnecessary referrals for images. Both groups cited time pressure as a factor in unwarranted imaging. As one GP stated, “*so instead of arguing with the patient for an hour about whether they need it or not, you write a referral in two minutes, and you’re done*.” While the majority GPs complained of not having enough time to relay information to patients, and ended up making a referral, radiologists were under the most time pressure in the assessment of numerous referrals.

## Discussion

Radiologists and GPs seem to have many similar perception regarding general barriers and facilitators for guideline adherence. These are, among others, the documents being too extensive and hard to find, and recommendations being perceived as either to strict or to ambiguous, and there being too many guidelines in total, as well as lack of time to read guidelines. This is consistent with earlier findings such as lack of time being a barrier to guideline use [9, 13, 25], the fear of loss of clinical autonomy, and ambiguous guidelines being less likely to be followed [26, 32, 33].

It is interesting that these barriers are still being reported, despite the general efforts being made to shorten guidelines, distributing guidelines to increase awareness and the perception that guidelines are strict standard that needs to be followed rigidly, and in all cases [34]. This perception can lead to skepticism towards the guidelines, resistance towards adhering to guidelines, and the feeling of loss of clinical autonomy. The fact that these barriers are still being reported highlights the importance of having a well-planned dissemination and implementation strategy when guidelines are developed, and adaption of these strategies towards the needs of the intended users.

However, this study shows that lack of awareness of the guideline is not as important a barrier for guideline use as earlier reported since most GPs reported having at least heard of the national guideline for diagnostic imaging in non-traumatic musculoskeletal diseases (5/8 GPs). However, only two GPs reported having tried to use the guideline. This indicates that other barriers for guideline use, such as lack of time, are more important in this setting.

The general facilitators reported are easy accessibility, guidelines being short, the knowledge basis is clear and agreed with, and the guidelines is perceived as being adapted towards the target group. One respondent also mentioned Computerized Physician Order Entry (CPOE) as a potential facilitator. These findings corresponds with earlier literature [13]. However, where this study differs from the earlier reports are that the importance of leaders as a facilitators for guideline use not being as emphasized [24].

The most important facilitator for the use of guidelines reported in this study were easy access to the guidelines, either digitally from the Norwegian Clinical Manual (NEL), which is the most used digital resource by the GPs, or as booklets, posters or one sheet of A4 paper that can be kept at the workstation. NEL is a general medical encyclopedia that is targeted at doctors and other health care professionals. Although the importance of leaders was not emphasized as a facilitator for guideline use by our participants, leadership and organization was mentioned as a factor that could affect the amount of unwarranted imaging being performed in the hospitals.

In our study we also found a larger difference in what they perceive as barriers to and facilitators of guideline adherence than earlier reported; where it was found that most of the barriers and facilitators perceived by GPs and radiologists were similar [26]. The differences seen both in barriers and facilitators for guideline use, and factors contributing to unwarranted imaging could be related to profession.

In our study radiologists perceived guidelines as less useful than the GPs, and were more insistent that they be developed by the right authorities, who are familiar with the setting in which they will be used. They regarded protocols and knowledge sharing more useful than national guidelines. This may be because they perceive guidelines are outdated by the time they are published, but may also indicate a lack of trust in guidelines or failing to recognize their value. It may also be that radiologists tend to disagree more with guidelines regarding radiology than GPs. Lastly, it may also be related to that radiologists may be more familiar with protocols than guidelines, and this unfamiliarity with guidelines making it harder to recognize their value.

Factors contributing to unwarranted imaging were reported as an increased demand and expectation of diagnostic imaging being performed, heavier pressure for documentation of disease as well as ruling out disease. Both radiologists and GPs also perceived diagnostic imaging as very useful for diagnosing musculoskeletal disease, and increasing patient safety. More controversial contributing factors are political and economic incentives (such as organizational structure and privatization) in performing diagnostic imaging, which the radiologists stressed. This is also consistent with the literature [35–38].

Privatization of health care was a key factor in unwarranted imaging according to both groups. This was seen as health business rather than health care, where private facilities were more likely than public hospitals to overuse imaging. This was linked to the accessibility of the services, which again increased the use of diagnostic imaging, and thereby the amount of unwarranted imaging. It was viewed that private radiological institutions were less likely to decline referrals, even if the examination could be seen as unjustified, because they were dependent on production to make a profit. This was viewed as a problem especially by the radiologists, but also to some degree by the GPs. Since we only interviewed radiologists in a public hospital setting this view may be skewed. At the same time, public hospitals and medical centers are partly paid through production of services such as imaging, and radiologist viewed this demand for production in the public setting as an important factor in unwarranted imaging.

Radiologists also perceived organizational structure as a crucial factor influencing use, mostly related to the hospital’s capacity and the need to keep patients moving through the system. This differs from previous studies, where organizational structure has been associated with a lack of resources, equipment space, staff and financial resources [13]. This can be interpreted both as a barrier to guideline use, and as an influencing factor for unwarranted imaging, indicating a link between the two. Another factor that is seen both as a barrier to guideline use and a factor affecting unwarranted imaging is lack of time. It was reported that there were little time for discussions about what the optimal examination for a patient may be between the radiologist and referrer. This in combination with the increased demand and expectation from the patient, and the desire to rule out disease leads to only partly justified, or unwarranted imaging being performed.

Unique to this study we find that there is a relationship between the factors influencing unwarranted imaging, and the facilitators and barriers for guideline use. This implies that increased guideline adherence by removing barriers may reduce the amount of unwarranted imaging, and likely the amount of diagnostic imaging in total.

Further research regarding, and development and implementation of guidelines, should therefore focus on all levels (individual, organization and nation) where this is possible to lead to significant changes. In the future, there should also be an increased focus on using an educational or combinational approach when implementing guidelines, for example combining education and feedback on the amount of diagnostic imaging referred to or used. The main themes found in this study could also be used in future research for the development of a survey.

### Limitations

Like all empirical studies, this study has its limitations, in terms of sample size and the inclusion of professional groups. A larger sample size would strengthen the study and increase the possibilities for generalizing. Moreover, a larger study that covered more than two hospitals and 7 GP offices, or had more participants might have yielded different results. Furthermore, we have limited our sample to GPs and radiologist. The inclusion other health care workers who can make referrals to diagnostic imaging, such as chiropractors and orthopedic surgeons, might also have produced different results.

In addition, we do not have specific age data for the participants, and this may skew the results, since significant difference in the age distribution of the two groups could alter their viewpoints or perceptions. We also lack data on time practicing for the participants, which could also show differences in attitudes correlating to how long they have practiced.

Lastly, it can be seen as a limitation that the use of CPOE as a tool for guideline implementation were not specifically addressed in the interviews, since this already is, and increasingly will be, used as such.

## Conclusions

In conclusion, we identified a wide range of barriers (guidelines being too long and hard to read, hard to find) and facilitators (easy access, adapted to the target group) for guideline adherence, as well as a wide range of factors affecting unwarranted imaging (time pressure, pressure from patients). Several of these factors coincide, indicating that increased adherence to relevant guidelines for both referrers and executers of diagnostic imaging will reduce the amount of unwarranted imaging. There were also found that radiologists and GPs perception of barriers and facilitators were somewhat similar (easy access and lack of time), and that there were some differences (radiologists perceived guidelines as less useful and more outdated).

Based on our results, we recommend that development and implementation of guidelines are followed by elaborated educational elements to inform future users, as publishing or postal dissemination clearly is not an efficient way to make users apply new guidelines. Governments should be clearer in their information of the guidelines, that they are recommendations for care, rather than standards.

Moreover guidelines should be integrated into the electronic systems that GPs use during the patient consultation and decision aid, such as a CPOE, electronic handbooks or other resources frequently used by the target group for the guideline. Last, but not least, a short version of maximum two pages should be developed for easy access to the key messages of the guideline at all times. These points should be supported by targeted education adapted to the intended users and their local environment, preferably in existing educational settings, in cooperation with local opinion leaders.

## Additional file

Additional file 1: Interview guide translated from Norwegian to English. (PDF 116 kb)

## Abbreviations

CFIR: Consolidated Framework for Implementation Research
CPOE: Computerized Physician Order Entry
GP: General Practitioner
MSDs: Musculoskeletal Diseases
NAV: Norwegian Labour and Welfare Services
NEL: Norwegian Clinical Manual
NMSG: Norwegian Musculoskeletal Guideline
NSD: Norwegian Social Science Data Services
