# Supplementary material for: Barriers and facilitators for guideline adherence in diagnostic imaging: an explorative study of GPs’ and radiologists’ perspectives
Source: BMC Health Serv Res. 2018 Jul 16;18:556. doi: 10.1186/s12913-018-3372-7 (PMC6048703; doi:10.1186/s12913-018-3372-7)
Supplement: Supplementary file 1 — Interview guide translated from Norwegian to English. (PDF 116 kb) [file 12913_2018_3372_MOESM1_ESM.pdf]

Interview guide

Knowledge of the guideline

- Can you tell me what you know of the Norwegian Musculoskeletal guideline?
- Additional questions:
  - Have you heard of the guideline?
  - Do you know where you can find the guideline?
  - Explain the guideline shortly if they have not heard of it before
- Have you used the guideline before? Why/why not?

Barriers and facilitators for guideline adherence

- What do you feel characterizes a good guideline?
  - Why are these factors important for you?
  - Are there any additional factors that make it more likely for you to use/adhere to a guideline?
- What do you feel characterizes a bad guideline?
  - Why are these the characterizations of a bad guideline for you?
  - Are there any additional factors that make it less likely for you to use/adhere to a guideline?

Guideline implementation

- Which implementation strategies have you experienced that have worked for you?
  - What was it about these strategies that worked for you?
  - If there are no strategies that have been experienced to work: why have not the strategies tried not worked for you?
- Use one or more examples of strategies tried earlier – Do you think this kind if implementation strategy would have worked for you?
  - Why / or why not?
- Are there any other implementation methods that you think could have worked for you?

Use of radiological services

- What do you feel affects the way radiological services are used today?
  - Follow up questions as needed
